# Association of Regional Practice Environment Intensity and the Ability of Internists to Practice High-Value Care After Residency

**DOI:** 10.1001/jamanetworkopen.2020.2494

**Published:** 2020-04-10

**Authors:** Weifeng Weng, Jessica Van Parys, Rebecca S. Lipner, Jonathan S. Skinner, Brenda E. Sirovich

**Affiliations:** 1 American Board of Internal Medicine, Philadelphia, Pennsylvania; 2 Department of Economics, Hunter College, New York, New York; 3 Dartmouth Institute for Health Policy & Clinical Practice, Hanover, New Hampshire; 4 Department of Economics, Dartmouth College, Hanover, New Hampshire; 5 Outcomes Group, Veterans Affairs Medical Center, White River Junction, Vermont

## Abstract

**Importance:**

Use of health care services and physician practice patterns have been shown to vary widely across the United States. Although practice patterns—in particular, physicians’ ability to provide high-quality, high-value care—develop during training, the association of a physician’s regional practice environment with that ability is less well understood.

**Objective:**

To examine the association between health care intensity in the region where physicians practice and their ability to practice high-value care, specifically for physicians whose practice environment changed due to relocation after residency.

**Design, Setting, and Participants:**

This cohort study included a national sample of 3896 internal medicine physicians who took the 2002 American Board of Internal Medicine initial certification examination followed approximately 1 decade (April 21, 2011, to May 7, 2015) later by the Maintenance of Certification (MOC) examination. At the time of the MOC examination, 2714 of these internists were practicing in a new region. Data were analyzed from March 6, 2016, to May 21, 2018.

**Exposures:**

Intensity of care in the Dartmouth Atlas hospital referral region (HRR), measured by per-enrollee end-of-life physician visits (primary) and current practice type (secondary).

**Main Outcomes and Measures:**

The outcome, a physician’s ability to practice high-value care, was assessed using the Appropriately Conservative Management (ACM) score on the MOC examination, measuring performance across all questions for which the correct answer was the most conservative option. The exposure, regional health care intensity, was measured as per-enrollee end-of-life physician visits in the Dartmouth Atlas HRR of the physician’s practice.

**Results:**

Among the 3860 participating internists included in the analysis (2030 men [52.6%]; mean [SD] age, 45.6 [4.5] years), those who moved to regions in the quintile of highest health care intensity had an ACM score 0.22 SD lower (95% CI, −0.32 to −0.12) than internists who moved to regions in the quintile of lowest intensity, controlling for postresidency ACM scores. This difference reflected scoring in the 44th compared with the 53rd percentile of all examinees. This association was mildly attenuated (0.18 SD less; 95% CI, −0.28 to −0.09) after adjustment for physician and practice characteristics.

**Conclusions and Relevance:**

This study found that practice patterns of internists who relocate after residency training appear to migrate toward norms of the new region. The demands of practicing in high-intensity regions may erode the ability to practice high-value conservative care.

## Key Points

**Question:**

How does the health care environment in a region influence internists’ clinical capabilities, particularly the ability to practice high-value care?

**Findings:**

This cohort study of 2714 newly certified internists (in 2002) who relocated to a new region after completing residency found that higher intensity of use of health care services in a physician’s destination region was associated with reduced ability to practice appropriately conservative care 1 decade later compared with that ability measured at the end of residency.

**Meaning:**

The demands of practicing in high-intensity service regions may erode internists’ ability to practice high-value, conservative care.

## Teaser

This cohort study examines the association between health care intensity in the region where physicians practice and their ability to practice high-value care, specifically for physicians who relocated after residency.

## Introduction

Health care reform efforts in the United States seek to improve quality of care while controlling costs and avoiding overuse of services.^1^ The success of such efforts depends critically on primary care physicians who, as frontline health care professionals, exert a disproportionate influence on clinical management and use of services.^2,3^ Prior research has identified the physician training environment as a potent influence on physicians’ capability to provide high-value care.^4,5,6,7,8,9^ However, little to no evidence is available on how this capability evolves when physicians relocate to a new practice environment after residency training.

Practicing high-value care often requires physicians to adopt less intensive management strategies, such as watchful waiting, less expensive interventions, or withdrawal of therapies, instead of using more costly medical interventions.^9^ In this study, we track physicians’ ability to provide high-value care over time, using the previously developed Appropriately Conservative Management (ACM) score. Derived from the American Board of Internal Medicine’s (ABIM) certifying examination, the ACM score measures the examinees’ ability to select the correct response when it is the most conservative management option presented. Assessment of clinical decision-making using written vignettes has been demonstrated to mirror similar assessments that use standardized patients^10^ to test clinically relevant scenarios.^11^

To study the evolution of this ability over time, we focused on a cohort of physicians who took the ABIM initial certification examination in 2002 for the first time and completed the Maintenance of Certification (MOC) examination approximately 1 decade later. In particular, we evaluated internal medicine graduates who were exposed to a new practice environment after completing residency training—that is, who relocated to a hospital referral region (HRR) different from that where they had trained.

## Methods

### Overview

We retrospectively followed the cohort of all physicians who took the initial ABIM internal medicine certification examination in 2002, until they took (or did not take) the MOC examination during the period from April 21, 2011, to May 7, 2015. Physicians were required to take and pass an MOC examination 10 years after passing their initial examination to stay certified. For each MOC examinee, we categorized the practice environment of the residency training program and that of their subsequent practice location (around the time of their MOC examination) based on measures of regional use of health care services. We examined the association between a physician’s ability to practice high-value care, measured by the ACM score, with health care intensity of the physician’s practice region, controlling for the ACM score on the initial certification examination, and further adjusting for physician and practice characteristics. We conducted a comparable analysis for the outcome of overall clinical competence, measured by the MOC overall score. This cohort study followed the Strengthening the Reporting of Observational Studies in Epidemiology (STROBE) reporting guideline.^12^ The project was approved by the institutional review board at Geisel School of Medicine at Dartmouth College, Hanover, New Hampshire. Because all Medicare data used in this study were publicly available on http://www.dartmouthatlas.org, the institutional review board deemed the study exempt from human participants review and informed consent.

### Study Sample

This study used the ABIM administrative database to define the study cohort and obtain physicians’ examination performance, training, demographic characteristics, and practice characteristics. A total of 6662 physicians who first took the internal medicine initial certification examination in 2002 and passed on the first or a subsequent attempt were identified. Physicians’ practice location zip code as of June 2016 was retrieved by matching their medical license with Centers for Medicare & Medicaid Services’ national plan and provider enumeration system.^13^ Most physicians (4812 [72.2%]) were practicing in a different HRR compared with where they had trained. Our cohort is based on the subset of 3896 physicians who subsequently took an MOC examination 9 to 13 years later (April 21, 2011, to May 7, 2015). Each practice location zip code was assigned to a Dartmouth Atlas practice HRR^14^; each residency program was also assigned to a training HRR based on the zip code of the residency training hospital. Of these, 2714 physicians were practicing in a different HRR compared with where they had trained, constituting the primary cohort for the study.

### Outcomes and Other Measures

The ABIM Internal Medicine MOC examination (2011-2015) consists of 180 single-best-answer questions. It emphasizes diagnosis and management of prevalent conditions for which clinical intervention can have important consequences for patients, focusing on areas in which practice has changed in recent years. Management questions (the majority) describe clinical scenarios and ask the examinee to use clinical judgment in selecting among management options.^15^

#### ACM Score

Our measure of practice style sought to evaluate physicians’ capability to pursue a management strategy with low levels of health care services when appropriate. Through a previously validated protocol,^9^ all management questions on the initial ABIM certification and MOC examinations were evaluated for potential inclusion in the ACM subscale. Only questions for which 2 trained coders (including B.E.S.) concurred that the correct response option represented a less intensive (costly) management strategy than all other incorrect response options—in other words, better care at lower cost—were included in the ACM subscale.

In 2002, all examinees took the same form of the initial ABIM Internal Medicine certification examination. The ACM score was therefore calculated as the proportion of ACM questions answered correctly and then standardized (mean [SD], 0 [1]). The MOC examinations (2011-2015), however, were administered across multiple forms. The ACM scores on the MOC examination were therefore scaled using the item-response theory model and then standardized (mean [SD], 0 [1]) to ensure that MOC scores were comparable across years and forms. A 1-SD improvement in ACM score is roughly equivalent to answering an additional 13% of the ACM questions correctly. Of 1438 unique questions tested in the 2011-2015 MOC examinations, 111 were ACM questions; a mean of 16 ACM questions were found per MOC examination.

#### Overall MOC Examination Performance

Overall performance on the MOC examination is considered to measure overall clinical competence in internal medicine. We used overall scores, normalized to a mean of 0 and an SD of 1, as a secondary outcome measure. A 1-SD improvement in overall score is roughly equivalent to answering an additional 8.7% of the examination questions correctly.

#### Individual Physician-Level Variables

Physician characteristics included sex, specialization (general internists or specialists, based on whether they received initial certification in an internal medicine specialty), international medical school graduate status, direct patient care full-time equivalent, and practice type (eg, solo or group). Prior research^9,16,17^ has shown that these characteristics are associated with internal medicine initial and MOC examination scores and practice performance measures.

#### Practice Environment

We measured practice environment intensity at the regional level by the mean number of physician visits within the last 6 months of life for fee-for-service Medicare beneficiaries 65 years or older residing within 1 of 306 HRRs; this measure has been used in other studies to measure health care intensity.^18,19,20^ Health care intensity was defined both for the training environment (based on a 20% sample of beneficiaries who died during 2001-2005) and for the subsequent practice environment (based on a 100% sample of beneficiaries who died in 2012). In sensitivity analyses, we used 2 alternative measures: mean total number of intensive care days per decedent in the last 6 months of life, and mean of total Medicare spending per enrollee (Parts A and B) adjusted for age, sex, race, and price. We used the logarithmic transformation of each of the 3 intensity measures to address potential skewness in the distributions of the measures.

### Statistical Analysis

Data were analyzed from March 6, 2016, to May 21, 2018. We used χ^2^ tests and 2-tailed *t* tests to compare the differences between physicians who relocated and who did not after residency training. Using regression analysis with the physician as the unit of analysis, we test the hypothesis that MOC examination performance is associated with intensity of the current practice environment. The primary exposure was intensity of the practice environment (the end-of-life visit index), and the primary outcome was practice style (ACM score); clinical competence (overall score) was included as a secondary outcome. The exposure and both outcome measures were modeled as continuous variables. To adjust for the clustering effect of physicians within HRRs, generalized estimating equation analysis with Huber-White standard error estimates was performed, controlling for performance on the certifying examination (ACM or overall score, respectively). Practice type was examined as a secondary exposure. The full model also controlled for physician characteristics; 95% CIs were calculated. A measure of intensity in the training HRR was not included in the model because the certifying examination was taken after completing residency training; thus, the training environment was already reflected in baseline scores.

For display purposes, HRRs were categorized into quintiles (with roughly equal numbers of physicians), based on the end-of-life visit index for 2012, with quintiles defined across the entire sample of physicians. Stata, version 14 (StataCorp LLC) was used for all statistical analysis. Two-sided *P* < .05 indicated significance.

## Results

### Movers and Stayers

**Figure 1.**
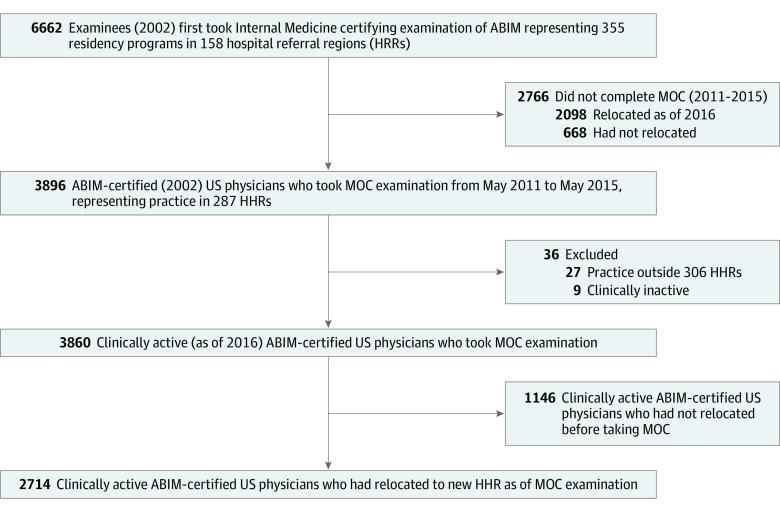
Flow Diagram of Identification of the Primary Internal Medicine Physician Cohort for the Study The cohort was selected based on timing of the initial Internal Medicine certification examination of the American Board of Internal Medicine (ABIM), completion of Maintenance of Certification (MOC) examination approximately 1 decade later, and current practice location. HHR indicates hospital referral region.

Among our cohort of 3896 physicians who took their initial certifying examination in 2002 and subsequently took an MOC examination, we excluded those not practicing in an HRR region (n = 36). This yielded a study sample of 3860 (1830 women [47.4%] and 2030 men [52.6%]; mean [SD] age, 45.6 [4.5] years); of these, 2714 physicians had moved to different HRRs after completing residency training (movers), whereas 1146 remained in the same HRR (stayers) (Figure 1). We also analyzed a subset of 2797 physicians who had both the destination practice zip code used in the analysis and a practice zip code from a different point in time after training but before taking the MOC examination, comparing HRR and the distance between the 2 practice locations. We found that 2593 physicians (92.7%) physicians remained in the same HRR or within 150 miles.

**Figure 2.**
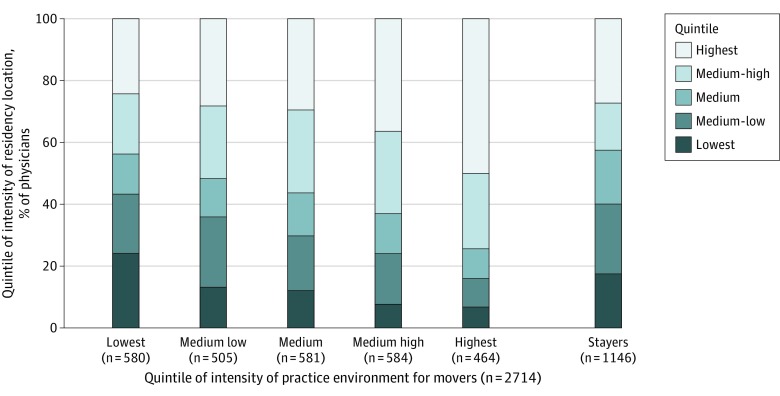
Relocation Patterns Among First-Time Internal Medicine American Board of Internal Medicine Certification Examinees (2002) Data are stratified according to quintile of care intensity of the destination hospital referral region (HRR) of the examinee’s practice location at the time of the Maintenance of Certification (MOC) examination. The HRRs, ordered according to the end-of-life visit index, were divided into quintiles of approximately equal number of physicians; practice and residency location HRR quintiles were categorized according to the same definition. Stayers indicate physicians who, at the time of the MOC examination, practiced in the same HRR in which they completed residency training.

Figure 2 shows relocation patterns of physicians according to quintile of health care intensity of their practice location. Among movers who practiced in the lowest-intensity quintile, for example, 140 of 580 (24.1%) had trained in a region in the lowest quintile and 141 (24.3%) had trained in a region in the highest quintile of health care intensity. For the highest-intensity quintile, just 32 of 464 physicians (6.9%) moved from a region in the lowest quintile, and 231 (49.8%) moved from a region in the highest quintile. The intensity measure had a correlation coefficient of 0.95 from the 2001-2005 period to 2012.

### Physician Characteristics

Of the 2714 movers, 1251 (46.1%) were female and 1268 (46.7%) were international medical school graduates. A total of 1342 movers (49.4%) were in a group practice; 238 (8.8%), a solo practice. One thousand five hundred fifty-four (57.3%) were general internists (ie, did not pursue a subspecialty certification) (Table). Stayers were more likely to be female (579 of 1146 [50.5%]), general internists (788 [68.8%]), and in academic practice (306 [26.7%]) and much less likely to be international medical school graduates (314 [27.4%]). As of June 2016, internists in the study practiced in 287 of 306 HRRs; residency training had taken place in 158 HRRs. Movers and stayers were not statistically different in initial scores, but different in MOC scores (mean [SD], −0.03 [1.00] for movers vs 0.06 [1.00] for stayers). Movers were trained in higher-intensity (mean [SD], 29.5 [9.9] visits) and practiced in lower-intensity (mean [SD], 36.6 [10.7] visits) HRRs compared with stayers (mean [SD], 34.4 [10.9] and 34.4 [11.2] visits, respectively) (Table). Box plots of scores and intensity measures are presented in eFigure 1 in the Supplement.

**Table.** Physician Demographic and Practice Characteristics

Characteristic	Physician Group^a^			*P* value^b^
	Full sample (n = 3860)	Movers (n = 2714)	Stayers (n = 1146)	
Female	1830 (47.4)	1251 (46.1)	579 (50.5)	.01
International medical school graduate	1582 (41.0)	1268 (46.7)	314 (27.4)	<.001
Practice type				
Solo	324 (8.4)	238 (8.8)	86 (7.5)	<.001
Academic	737 (19.1)	431 (15.9)	306 (26.7)	
Group	1843 (47.7)	1342 (49.4)	501 (43.7)	
Hospital inpatient	588 (15.2)	456 (16.8)	132 (11.5)	
Military or government	156 (4.0)	103 (3.8)	53 (4.6)	
Other	212 (5.5)	144 (5.3)	68 (5.9)	
Direct patient care full-time equivalent				
Part-time <50%	320 (8.3)	197 (7.3)	123 (10.7)	<.001
Part-time ≥50%	1990 (51.6)	1401 (51.6)	589 (51.4)	
Full-time	1550 (40.2)	1116 (41.1)	434 (37.9)	
General internists	2342 (60.7)	1554 (57.3)	788 (68.8)	<.001
Initial certification score, mean (SD)				
ACM	0.00 (1.00)	−0.01 (1.01)	0.03 (0.99)	.23
Overall	0.00 (1.00)	−0.01 (0.99)	0.00 (1.02)	.75
MOC score, mean (SD)				
ACM	0.00 (1.00)	−0.03 (1.00)	0.06 (1.00)	.01
Overall	0.00 (1.00)	−0.03 (0.99)	0.06 (1.03)	.01
Environment intensity, No. of visits, mean (SD)				
Practice	30.0 (10.2)	29.5 (9.9)	31.3 (10.9)	<.001
Training	36.0 (10.9)	36.6 (10.7)	34.4 (11.2)	<.001

Abbreviations: ACM, Appropriately Conservative Management; MOC, Maintenance of Certification.

^a^ Unless otherwise indicated, data are expressed as number (percentage) of physicians. Percentages have been rounded and may not total 100.

^b^ Calculated from χ^2^ tests for the equality of percentages and 2-tailed *t* tests for the equality of means between movers and stayers.

### Association Between MOC Scores and Practice Environment

**Figure 3.**
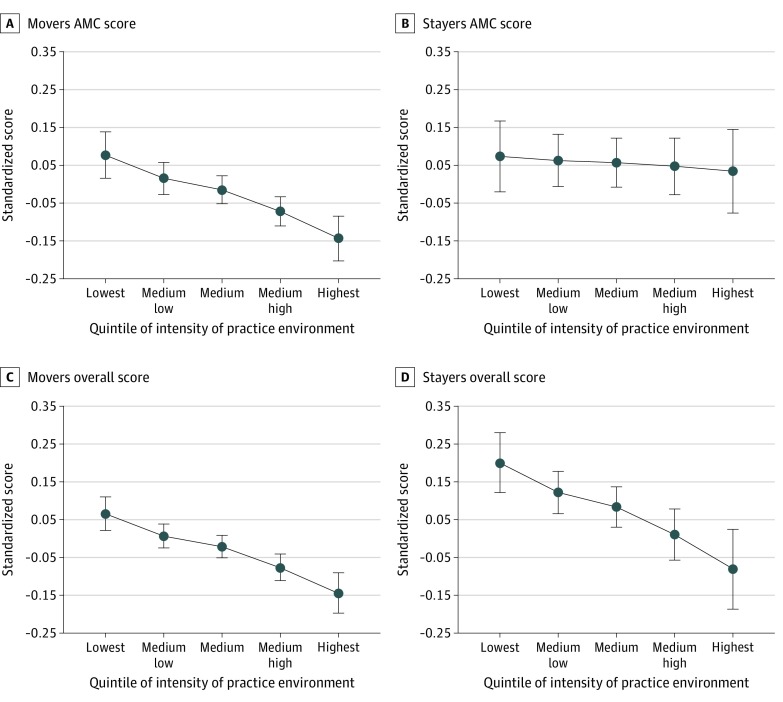
Association of Regional Care Intensity of Practice Location (Exposure) and Ability to Practice Conservatively (Primary Outcome) The hospital referral regions (HRRs), ordered according to end-of-life visit index, were divided into quintiles of approximately equal numbers of physicians; practice and residency location HRR quintiles were categorized according to the same definition. The primary outcome was measured by the Appropriately Conservative Management (ACM) score on the Maintenance of Certification (MOC) examination adjusted for the initial ACM score. Movers include 2714 internists who moved to a different HRR after completing residency training; stayers, 1146 internists who stayed in the same HRR where they completed residency training. Parts C and D display the same association for the secondary outcome, clinical competence (MOC overall score). All scores are standardized to a mean of 0 and an SD of 1. The standard score is reported at the midpoint of each quintile after adjusting for the score from the initial American Board of Internal Medicine certifying examination. Error bars indicate 95% CIs.

Figure 3 displays the practice style (ACM) score, conditional on initial score, displayed by quintile of health care intensity of the internist’s practice location (HRR). For movers, a pronounced negative gradient was found for the MOC ACM score across quintiles of increasing health care intensity. For example, conditional on the initial score, the MOC ACM score for the midpoint of the highest-intensity quintile (ie, 90th percentile of intensity) was 0.22 SDs less than that of the lowest-intensity quintile (ie, 10th percentile; 95% CI, −0.32 to −0.12) (Figure 3A), reflecting scoring in the 44th compared with the 53rd percentile of all examinees. For stayers, no difference was observed (Figure 3B). Overall scores were negatively associated with intensity of the practice environment for movers (Figure 3C) and stayers (Figure 3D), with the MOC overall score of physicians practicing in the highest-intensity quintile being 0.21 SD lower than that of physicians in the lowest-intensity quintile for movers (95% CI, −0.29 to −0.14).

### Multivariable Findings

**Figure 4.**
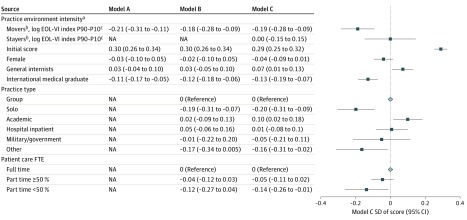
Association Between Ability to Practice Conservatively (Appropriately Conservative Management [ACM] Score) and Care Intensity, Physician Characteristics, and Practice Characteristics All models are adjusted for ACM score of the initial certification examination for internal medicine. Model A includes movers only, with care intensity and physician characteristics; model B, movers only, with all covariates; and model C, movers and stayers. Results of the full model (C) are represented in the graph. NA signifies not applicable. ^a^Measured at the level of each of the Dartmouth Atlas’s 306 hospital referral regions as the mean total number of physician visits per decedent within the last 6 months of life in the year 2012. ^b^For model C, a stayers indicator and an interaction term between intensity and stayers indicator were included in the model. Practice environment intensity for stayers and movers report the combined estimated effect of intensity and the interaction term between intensity and stayers indicator for stayers and movers, respectively. ^c^Reflects the change in end-of-life visit index (EOL VI) score based on the difference between the 90th and 10th percentiles of intensity measure.

For movers, the negative gradient of MOC ACM scores with regard to the practice environment changed minimally after adjustment for physician characteristics (Figure 4, model A). Adjusted ACM score in the midpoint of the highest-intensity quintile (90th percentile) was 0.21 SD less than that of the midpoint of the lowest-intensity quintile (ie, 10th percentile; 95% CI, −0.31 to −0.11). Further adjustment for practice characteristics modestly attenuated the effect; adjusted ACM score in the highest-intensity quintile was 0.18 SD less than that in the lowest-intensity quintile (95% CI, −0.28 to −0.09), reflecting scoring in the 45th compared with the 52nd percentile of all examinees. Physicians’ MOC overall scores exhibited a similar pattern; the adjusted overall score in the highest-intensity quintile was 0.15 SD less than that in the lowest-intensity quintile (95% CI, −0.22 to −0.07) (eFigure 2 in the Supplement).

Practice type also had an important association with the MOC score (Figure 4, model B). Compared with physicians in group practice, solo practitioners scored 0.19 SDs less (95% CI, −0.31 to −0.07) for the ACM score; similar results were obtained for the overall score.

Finally, we conducted sensitivity analysis by including movers and stayers (Figure 4, model C). For stayers, the MOC ACM score was not associated with intensity of the practice environment (the difference between the adjusted ACM score in the highest intensity quintile and in the lowest quintile was 0.0 SD [95% CI, −0.15 to 0.15]), whereas the MOC overall score exhibited a similar pattern as for movers (the adjusted overall score in the highest intensity quintile was 0.20 SD less that in the lowest-intensity quintile [95% CI, −0.31 to −0.08]) (eFigure 2, model C in the Supplement). We also repeated regression analyses of practice style and clinical competence using 2 alternative environment intensity measures: end-of-life intensive care index and per enrollee Medicare spending (eTable 1 and eTable 2 in the Supplement), with very similar results. In the third sensitivity analysis, we constructed a knowledge score that removed the ACM and other management questions and then repeated the regression analyses, with very similar results to the overall score models (eTable 3 in the Supplement).

## Discussion

In our study of the association between physician practice environments and their clinical skills approximately 1 decade after residency, we tested the influence of the practice environment independent of residency training. We found that high-intensity practice environments are negatively associated with the capability of physicians to practice conservatively when clinically indicated and overall clinical competence, even after adjusting for the physicians’ initial examination scores. Practice microenvironments (ie, organizational structure) were also significantly associated with scores.

One advantage of our approach is that we have sidestepped difficulties inherent in risk adjustment.^21^ Our measures of appropriately conservative management and clinical competence are independent of the health of patients and do not require case-mix adjustment. Furthermore, the physicians taking the examination have a very strong incentive to answer the questions carefully and to the best of their knowledge.

Our results are consistent with prior evidence^22^ suggesting that the intensity of the residency-training environment affects physicians’ ability to practice conservatively but that these effects may decay over time, perhaps as the current practice environment comes to exert a stronger influence on practice style.^16,23,24,25,26^ As well, previous literature has found evidence that physicians in regions with higher levels of health expenditures are more likely to recommend treatments without evidence of effectiveness^27,28^ and that solo practitioners may lag behind with regard to new clinical developments.^17,29,30^ For stayers, high-intensity practice environments are not associated with ACM score after adjusting for their initial ACM score, suggesting that the ability to practice conservatively (or not) may be reinforced by the consistency of the practice environment. For movers and stayers, high-intensity practice environments were associated with lower overall clinical competence after adjusting for the physicians’ initial score, suggesting overall clinical competence may respond to peer effects through professional and social interactions.

What is the mechanism by which health care intensity affects ABIM scores? Previous research has suggested that a more intense practice environment is associated with worse physician satisfaction,^19,31^ and time spent coordinating care is potentially stressful for health care professionals.^32^ Although there are mixed associations between intensity of care and quality,^19,20,33,34^ researchers find that poor physician evaluation and management (eg, higher preventable hospital admission rates) are more often associated with higher health care intensity.^33^ This nexus of satisfaction, practice environment, and quality of care is at least consistent with the view that physicians whose patients require more intensive and complex treatments have less time and energy to invest in professional development.

Previous research has also suggested that a more intense practice environment is associated with greater use of specialty care, more fragmented care, and more frequent ordering of diagnostic tests and minor procedures.^19^ In the high-intensity environment, physicians could become more dependent on specialist referrals and less experienced with their own clinical management capabilities, with potentially adverse effects on subsequent MOC examination scores. However, we cannot rule out other mechanisms; for example, MOC scores may matter more for job placement and retention or for attracting and retaining patients in low-intensity regions.

Several policy implications are associated with preserving clinical competence and supporting the ability to practice appropriately conservative management. First, the negative association of HRR intensity with appropriately conservative practice style and clinical competence underlines a potential positive feedback loop between reducing health care intensity and retaining and enhancing appropriately conservative practice style and clinical competence. One straightforward approach would be to inform physicians of their ACM scores (in addition to their overall clinical competence scores), making clear where they stand relative to their peers. Specific resources or training could be made available to those with subpar scores. In addition, recognizing that physician burnout in high-intensity practice environments could have long-term effects on erosion in conservative practice style and knowledge acquisition could help to motivate improved time management in such practices.

Second, our results suggest that practice type matters; physicians in solo practices and physicians working with patients part-time generally fare worse than those working full-time in groups. Because independent practice associations can bring together a large number of physicians relatively quickly and inexpensively, in addition to their traditional functions as contracts negotiators, they could also provide physicians the opportunities to participate in initiatives such as accountable care organizations and quality improvement programs.

### Limitations

Our study has several limitations. First, the primary measure of regional health care intensity is an ecological measure. However, this type of measure has been used in other studies to capture regional intensity of care.^9,35^ Because patients may self-select at the practice level, an analysis based on practice-level use would be subject to self-selection confounding. In addition, our results are robust to different types of intensity measures. Second, the outcome measures, practice style and clinical competence, are based on responses to questions on the internal medicine MOC examination, a simulated clinical practice environment, rather than clinical decisions made in direct patient care. However, prior work has established a direct association between performance on certification examinations and subsequent practice performance.^9^ Furthermore, the questions asked in the ABIM examinations are strongly associated with the types of diagnoses and patients encountered in clinical practice.^11^

Third, we only used the training and destination HRRs, not HRRs where intermediate practice was located. Because midcareer moving is quite costly for physicians in terms of medical licensure and other costs, it happens infrequently; 92.7% of our sample who had a practice location from a different point after training available remained either within 150 miles of their initial location or within the same HRR. In addition, the intensity measure was very stable, with a correlation coefficient of 0.95 from the 2001-2005 period to 2012.

Fourth, our study was limited to internists who took the internal medicine MOC examination 10 years later. The results might not be generalizable to those who did not return for the MOC. However, historically, about 80% of ABIM-certified general internists and 50% of internal medicine specialists take the internal medicine MOC examination. Specialists have a lower rate of return because they typically no longer practice general internal medicine and are not required to take the internal medicine MOC examination to maintain their specialty certificate. Therefore, our study cohort represented most internists who practice general internal medicine.

Fifth, this is an association study; the effects of the intensity of the practice environment and practice type might be partially the result of self-selection. Although we adjusted for initial certification score, we acknowledge that other unmeasured confounding could affect practice location choices and changes in physicians’ examination scores.

## Conclusions

Using a cohort of physicians who first took their ABIM certifying examination in 2002, we considered factors that were associated with their ACM scores and overall clinical competency approximately 1 decade later. We found the intensity of care at the regional level was negatively associated with both capability of physicians to practice conservatively when clinically indicated and overall clinical competence. Several other factors, such as solo vs group practice and part-time vs full-time practice, were also negatively associated. These findings suggest that the goal of higher-quality health care at lower costs will be most effective when it takes into consideration improvements in the practice environment, whether by encouraging physicians to practice in groups or by changing practice styles and potentially unwarranted use of health care services in high-intensity regions.

## Notes

Supplement. **eFigure 1.** Distribution of Initial and MOC Examination Scores and Practice Environment Intensity **eFigure 2.** Association Between Regional Care Intensity of Practice Location (Exposure) and Clinical Competence, Measured by Overall Score on the Maintenance of Certification (MOC) Examination **eTable 1. **Association Between Regional Care Intensity of Practice Location (Exposure) and Conservative Practice Style and Clinical Competence: Alternative Measure 1—End-of-Life Intensive Care Index **eTable 2.** Association Between Regional Care Intensity of Practice Location (Exposure) and Conservative Practice Style and Clinical Competence: Alternative Measure 2—per Enrollee Medicare Spending **eTable 3.** Association Between Regional Care Intensity of Practice Location (Exposure) and Clinical Knowledge, Measured by Knowledge Score on the Maintenance of Certification (MOC) Examination
