# Supplementary material for: Association of Regional Practice Environment Intensity and the Ability of Internists to Practice High-Value Care After Residency
Source: JAMA Netw Open. 2020 Apr 10;3(4):e202494. doi: 10.1001/jamanetworkopen.2020.2494 (PMC7148442; doi:10.1001/jamanetworkopen.2020.2494)
Supplement: Supplement. — eFigure 1. Distribution of Initial and MOC Examination Scores and Practice Environment Intensity eFigure 2. Association Between Regional Care Intensity of Practice Location (Exposure) and Clinical Competence, Measured by Overall Score on the Maintenance of Certification (MOC) Examination eTable 1. Association Between Regional Care Intensity of Practice Location (Exposure) and Conservative Practice Style and Clinical Competence: Alternative Measure 1—End-of-Life Intensive Care Index eTable 2. Association Between Regional Care Intensity of Practice Location (Exposure) and Conservative Practice Style and Clinical Competence: Alternative Measure 2—per Enrollee Medicare Spending eTable 3. Association Between Regional Care Intensity of Practice Location (Exposure) and Clinical Knowledge, Measured by Knowledge Score on the Maintenance of Certification (MOC) Examination [file jamanetwopen-3-e202494-s001.pdf]

Supplementary Online Content

Weng W, Van Parys J, Lipner RS, Skinner JS, Sirovich BE. Association of regional practice environment intensity and the ability of internists to practice high-value care after residency. *JAMA Netw Open*. 2020;3(4):e202494. doi:10.1001/jamanetworkopen.2020.2494

eFigure 1. Distribution of Initial and MOC Examination Scores and Practice Environment Intensity
eFigure 2. Association Between Regional Care Intensity of Practice Location (Exposure) and Clinical Competence, Measured by Overall Score on the Maintenance of Certification (MOC) Examination
eTable 1. Association Between Regional Care Intensity of Practice Location (Exposure) and Conservative Practice Style and Clinical Competence: Alternative Measure 1—End-of-Life Intensive Care Index
eTable 2. Association Between Regional Care Intensity of Practice Location (Exposure) and Conservative Practice Style and Clinical Competence: Alternative Measure 2—per Enrollee Medicare Spending
eTable 3. Association Between Regional Care Intensity of Practice Location (Exposure) and Clinical Knowledge, Measured by Knowledge Score on the Maintenance of Certification (MOC) Examination

This supplementary material has been provided by the authors to give readers additional information about their work.

©2020 Weng W et al. *JAMA Network Open*.

eFigure 1. Distribution of Initial and MOC Examination Scores and Practice Environment Intensity

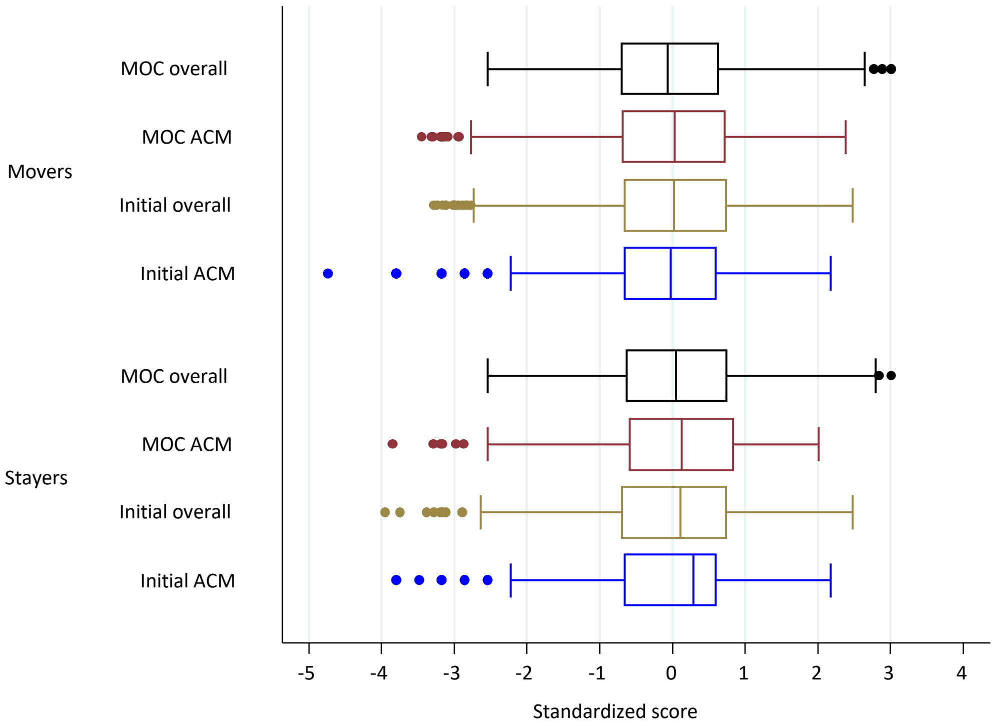

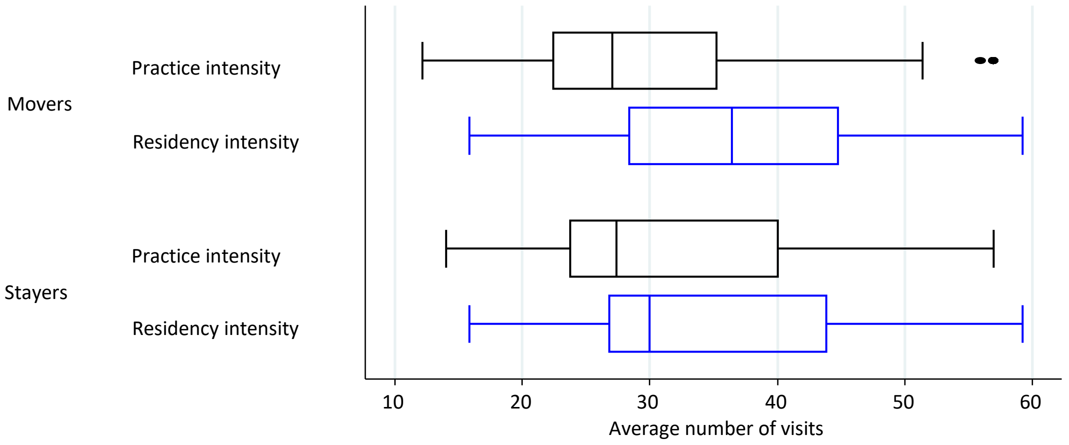

Practice intensity is based on physicians' practice locations and measured at the level of each of the Dartmouth Atlas's 306 Hospital Referral Regions (HRR) as the mean of the total number of physician visits per decedent within the last 6 months of life in year 2012.

Residency intensity is based on physicians' residency training locations and measured at the level of each of the Dartmouth Atlas's 306 Hospital Referral Regions (HRR) as the mean of the total number of physician visits per decedent within the last 6 months of life in years 2001-2005.

©2020 Weng W et al. *JAMA Network Open*.

eFigure 2. Association Between Regional Care Intensity of Practice Location (Exposure) and Clinical Competence, Measured by Overall Score on the Maintenance of Certification (MOC) Examination

All models adjust for overall score of the initial certification exam. Model A: Movers only, with care intensity and physician characteristics; Model B: Movers only, with all convariates; Model C: Movers + Stayers. Results of the full model (C) are represented in the figure.

	Model A: w/ MD traits (movers)	Model B: w/ practice traits (movers)	Model C: (movers +stayers)
Practice Environment Intensity[a] (movers)[b] log EOL-VI index P90-P10[c]	-0.19 (-0.26, -0.11)	-0.15 (-0.22, -0.07)	-0.14 (-0.22, -0.07)
Practice Environment Intensity (stayers)[b] log EOL-VI index P90-P10[c]	-	-	-0.20 (-0.31, -0.08)
Initial score	0.71 (0.68, 0.73)	0.70 (0.67, 0.73)	0.70 (0.67, 0.72)
Female	-0.08 (-0.15, -0.02)	-0.10 (-0.16, -0.04)	-0.09 (-0.14, -0.04)
General internists	0.12 (0.07, 0.18)	0.11 (0.05, 0.16)	0.12 (0.07, 0.18)
International medical graduate	-0.23 (-0.28, -0.17)	-0.23 (-0.28, -0.17)	-0.21 (-0.26, -0.15)
Practice Type			
Group		0 (referent)	0 (referent)
Solo		-0.22 (-0.33, -0.11)	-0.29 (-0.38, -0.19)
Academic		0.15 (0.06, 0.23)	0.21 (0.15, 0.28)
Hospital inpatient		0.16 (0.08, 0.24)	0.12 (0.06, 0.19)
Military/gov't		0.10 (-0.04, 0.25)	0.09 (-0.02, 0.21)
Other		0.04 (-0.09, 0.17)	0.02 (-0.09, 0.13)
Patient care FTE			
Full time		0 (referent)	0 (referent)
Part time ≥ 50%		-0.05 (-0.11, 0.01)	-0.05 (-0.1, 0.01)
Part time < 50%		-0.12 (-0.24, -0.01)	-0.15 (-0.25, -0.05)

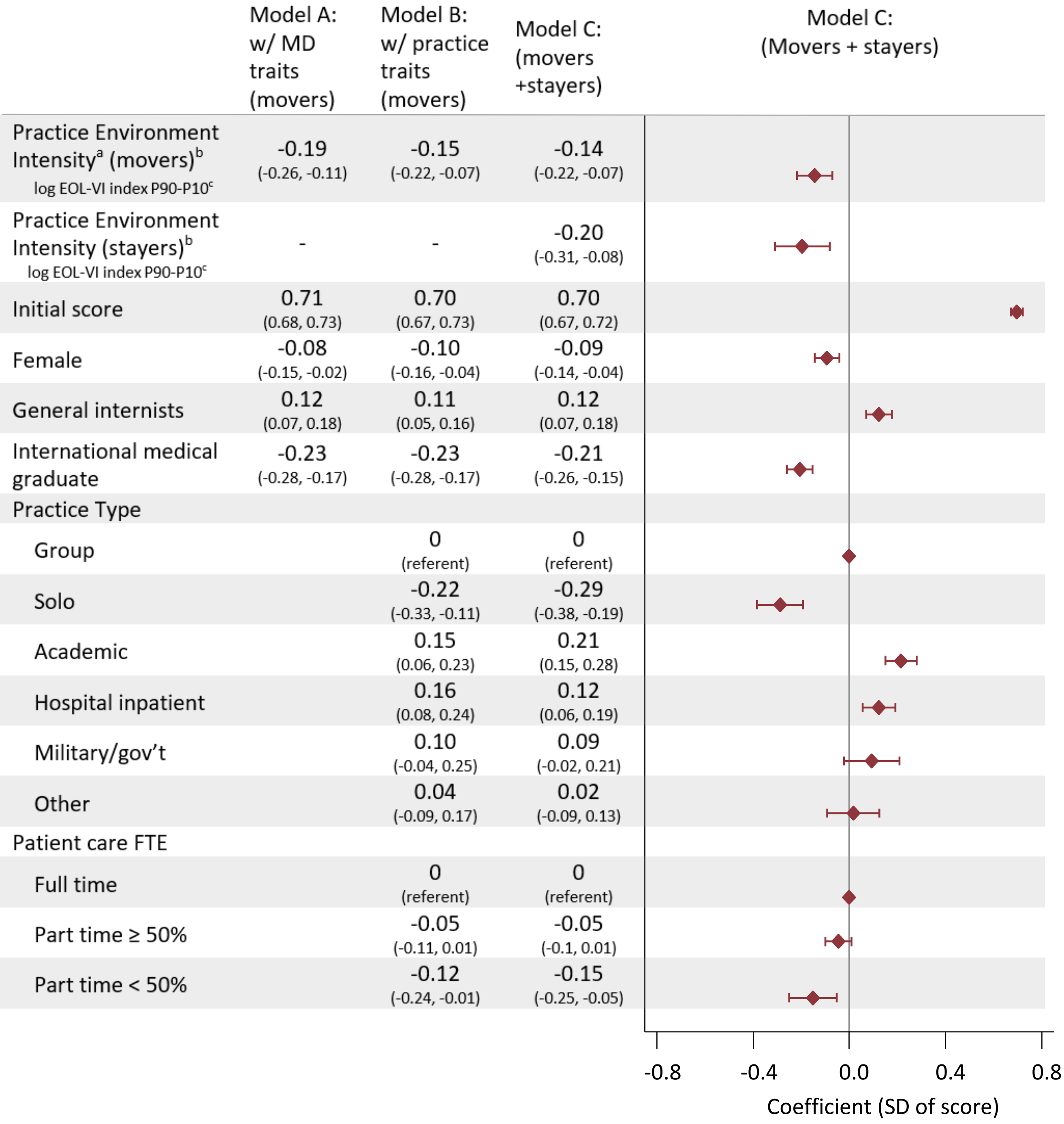

©2020 Weng W et al. *JAMA Network Open*.

[a]Practice Environment Intensity is measured at the level of each of the Dartmouth Atlas's 306 Hospital Referral Region (HRR) as the average total number of physician visits per decedent within the last 6 months of life in year 2012.
[b] For Model C (movers + stayers), a "stayers" indictor and an interaction term between intensity and stayers indicator were included in the model. "Practice environment intensity (stayers)" & "Practice environment intensity (movers)" report the combined estimated effect of intensity and the interaction term between intensity and stayers indicator for stayers and movers, respectively.
[c]P90-P10 reflects the change in score based on the difference between 90th – 10th percentile of intensity measure.

©2020 Weng W et al. *JAMA Network Open*.

eTable 1. Association Between Regional Care Intensity of Practice Location (Exposure) and Conservative Practice Style and Clinical Competence: Alternative Measure 1—End-of-Life Intensive Care Index

	Appropriately Conservative Practice Style (MOC ACM score)			Clinical Competence (MOC overall score)	
	Movers only	Movers + Stayers		Movers only	Movers + Stayers
Practice Environment Intensity[a] (movers)[b] log EOL-VI index P90-P10[c]	-0.18 (-0.28, -0.09)	-0.18 (-0.28, -0.09)		-0.10 (-0.17, -0.04)	-0.10 (-0.17, -0.03)
Practice Environment Intensity (stayers)[b] log EOL-VI index P90-P10[c]	-	0.09 (-0.05, 0.23)		-	-0.06 (-0.16, 0.05)
Initial ACM score	0.30 (0.26, 0.34)	0.29 (0.25, 0.32)		0.70 (0.67, 0.73)	0.70 (0.67, 0.72)
Female *(v. male)*	-0.02 (-0.10, 0.05)	-0.04 (-0.09, 0.02)		-0.1 (-0.16, -0.04)	-0.09 (-0.14, -0.04)
General internists	0.03 (-0.05, 0.1)	0.07 (0.01, 0.13)		0.11 (0.05, 0.16)	0.13 (0.07, 0.18)
International medical graduate	-0.12 (-0.18, -0.06)	-0.13 (-0.18, -0.07)		-0.23 (-0.28, -0.17)	-0.21 (-0.26, -0.16)
Practice Type					
Group	0 (referent)	0 (referent)		0 (referent)	0 (referent)
Solo	-0.19 (-0.31, -0.08)	-0.21 (-0.31, -0.1)		-0.23 (-0.34, -0.12)	-0.3 (-0.39, -0.2)
Academic	0.02 (-0.09, 0.13)	0.09 (0.01, 0.18)		0.15 (0.06, 0.23)	0.21 (0.15, 0.28)
Hospital inpatient	0.05 (-0.05, 0.16)	0.01 (-0.08, 0.1)		0.17 (0.08, 0.25)	0.13 (0.06, 0.2)
Military/government	-0.01 (-0.22, 0.2)	-0.05 (-0.21, 0.11)		0.11 (-0.04, 0.25)	0.1 (-0.02, 0.21)
Other	-0.16 (-0.34, 0.01)	-0.16 (-0.31, -0.02)		0.04 (-0.09, 0.17)	0.01 (-0.09, 0.12)
Time in patient care					
Full time	0 (referent)	0 (referent)		0 (referent)	0 (referent)
Part time ≥ 50%	-0.04 (-0.12, 0.04)	-0.05 (-0.11, 0.02)		-0.05 (-0.11, 0.02)	-0.04 (-0.10, 0.01)
Part time < 50%	-0.12 (-0.27, 0.04)	-0.14 (-0.26, -0.01)		-0.12 (-0.24, -0.01)	-0.15 (-0.25, -0.05)

[a]Practice Environment Intensity is measured at the level of each of the Dartmouth Atlas's 306 Hospital Referral Region (HRR) as the mean of the total number of intensive care days per decedent in the last 6 months of life for Medicare beneficiaries aged 65 years or older in year 2012.

[b] For Model C (movers + stayers), a "stayers" indictor and an interaction term between intensity and stayers indicator were included in the model. "Practice environment intensity (stayers)" & "Practice environment intensity (movers)" report the combined estimated effect of intensity and the interaction term between intensity and stayers indicator for stayers and movers, respectively.

[c]P90-P10 reflects the change in score based on the difference between 90th – 10th percentile of intensity measure.

©2020 Weng W et al. *JAMA Network Open*.

eTable 2. Association Between Regional Care Intensity of Practice Location (Exposure) and Conservative Practice Style and Clinical Competence: Alternative Measure 2—per Enrollee Medicare Spending

	Appropriately Conservative Practice Style (MOC ACM score)			Clinical Competence (MOC Overall score)	
	Movers only	Movers + Stayers		Movers only	Movers + Stayers
Practice Environment Intensity[a] (movers)[b] log EOL-VI index P90-P10[c]	-0.18 (-0.30, -0.06)	-0.18 (-0.31, -0.06)		-0.19 (-0.27, -0.11)	-0.19 (-0.26, -0.11)
Practice Environment Intensity (stayers)[b] log EOL-VI index P90-P10[c]	-	0.02 (-0.13, 0.18)		-	-0.13 (-0.23, -0.02)
Initial ACM score	0.30 (0.26, 0.34)	0.29 (0.25, 0.32)		0.7 (0.67, 0.73)	0.7 (0.67, 0.72)
Female *(v. male)*	-0.03 (-0.11, 0.04)	-0.05 (-0.1, 0.01)		-0.11 (-0.17, -0.04)	-0.1 (-0.15, -0.05)
General internists	0.03 (-0.05, 0.1)	0.07 (0.01, 0.13)		0.1 (0.05, 0.16)	0.13 (0.07, 0.18)
International medical graduate	-0.11 (-0.18, -0.05)	-0.13 (-0.18, -0.07)		-0.22 (-0.28, -0.17)	-0.21 (-0.26, -0.15)
Practice Type					
Group	0 (referent)	0 (referent)		0 (referent)	0 (referent)
Solo	-0.19 (-0.31, -0.07)	-0.20 (-0.31, -0.09)		-0.22 (-0.33, -0.11)	-0.29 (-0.39, -0.2)
Academic	0.03 (-0.08, 0.14)	0.10 (0.02, 0.18)		0.15 (0.07, 0.24)	0.22 (0.15, 0.28)
Hospital inpatient	0.06 (-0.05, 0.17)	0.02 (-0.07, 0.11)		0.17 (0.09, 0.25)	0.13 (0.06, 0.2)
Military/government	0 (-0.21, 0.2)	-0.05 (-0.21, 0.12)		0.11 (-0.04, 0.25)	0.1 (-0.02, 0.22)
Other	-0.17 (-0.34, 0)	-0.17 (-0.31, -0.02)		0.04 (-0.09, 0.17)	0.01 (-0.1, 0.12)
Time in patient care					
Full time	0 (referent)	0 (referent)		0 (referent)	0 (referent)
Part time ≥ 50%	-0.04 (-0.12, 0.04)	-0.04 (-0.11, 0.02)		-0.05 (-0.11, 0.02)	-0.04 (-0.1, 0.01)
Part time < 50%	-0.12 (-0.27, 0.04)	-0.14 (-0.27, -0.01)		-0.13 (-0.24, -0.01)	-0.15 (-0.25, -0.05)

[a]Practice Environment Intensity is measured at the level of each of the Dartmouth Atlas's 306 Hospital Referral Regions (HRR) as the mean of the total Medicare reimbursements per Medicare beneficiary aged 65 years or older (Parts A and B) adjusted for age, sex, race and price in year 2012.
[b] For Model C (movers + stayers), a "stayers" indictor and an interaction term between intensity and stayers indicator were included in the model. "Practice environment intensity (stayers)" & "Practice environment intensity (movers)" report the combined estimated effect of intensity and the interaction term between intensity and stayers indicator for stayers and movers, respectively.
[c]P90-P10 reflects the change in score based on the difference between 90th – 10th percentile of intensity measure.

©2020 Weng W et al. *JAMA Network Open*.

eTable 3. Association Between Regional Care Intensity of Practice Location (Exposure) and Clinical Knowledge, Measured by Knowledge Score on the Maintenance of Certification (MOC) Examination

	Clinical Knowledge (MOC Knowledge score)	
	Movers only	Movers + Stayers
Practice Environment Intensity[a] (movers)[b] log EOL-VI index P90-P10[c]	-0.15 (-0.23, -0.07)	-0.15 (-0.23, -0.07)
Practice Environment Intensity (stayers)[b] log EOL-VI index P90-P10[c]	-	-0.25 (-0.4, -0.11)
Initial ACM score	0.64 (0.61, 0.68)	0.65 (0.62, 0.67)
Female *(v. male)*	-0.09 (-0.15, -0.03)	-0.10 (-0.15, -0.05)
General internists	0.07 (0.01, 0.12)	0.10 (0.05, 0.15)
International medical graduate	-0.23 (-0.29, -0.17)	-0.22 (-0.28, -0.16)
Practice Type		
Group	0 (referent)	0 (referent)
Solo	-0.19 (-0.31, -0.07)	-0.24 (-0.34, -0.14)
Academic	0.15 (0.06, 0.24)	0.23 (0.15, 0.3)
Hospital inpatient	0.12 (0.03, 0.21)	0.08 (0.01, 0.16)
Military/government	0.15 (0, 0.29)	0.12 (0, 0.24)
Other	0.08 (-0.05, 0.21)	0.08 (-0.02, 0.19)
Time in patient care		
Full time	0 (referent)	0 (referent)
Part time ≥ 50%	-0.05 (-0.12, 0.02)	-0.03 (-0.09, 0.03)
Part time < 50%	-0.09 (-0.22, 0.04)	-0.13 (-0.23, -0.03)

[a]Practice Environment Intensity is measured at the level of each of the Dartmouth Atlas's 306 Hospital Referral Region (HRR) as the average total number of physician visits per decedent within the last 6 months of life in year 2012.
[b]For Model C (movers + stayers), a "stayers" indictor and an interaction term between intensity and stayers indicator were included in the model. "Practice environment intensity (stayers)" & "Practice environment intensity (movers)" report the combined estimated effect of intensity and the interaction term between intensity and stayers indicator for stayers and movers, respectively.
[c]P90-P10 reflects the change in score based on the difference between 90th – 10th percentile of intensity measure.

©2020 Weng W et al. *JAMA Network Open*.
